# Specific Changes in Young Soccer Player's Fitness After Traditional Bilateral vs. Unilateral Combined Strength and Plyometric Training

**DOI:** 10.3389/fphys.2018.00265

**Published:** 2018-03-22

**Authors:** Rodrigo Ramirez-Campillo, Javier Sanchez-Sanchez, Oliver Gonzalo-Skok, Alejandro Rodríguez-Fernandez, Manuel Carretero, Fabio Y. Nakamura

**Affiliations:** ^1^Department of Physical Activity Sciences, Research Nucleus in Health, Physical Activity and Sport, Universidad de Los Lagos, Osorno, Chile; ^2^Research Group Planning and Assessment of Training and Athletic Performance, Pontifical University of Salamanca, Salamanca, Spain; ^3^Faculty of Health Sciences, University of San Jorge, Zaragoza, Spain; ^4^Facultad de Ciencias de la Salud, Universidad Isabel I, Burgos, Spain; ^5^The College of Healthcare Sciences, James Cook University, Townsville, QLD, Australia; ^6^Department of Medicine and Aging Sciences, “G. d'Annunzio” University of Chieti-Pescara, Chieti, Italy

**Keywords:** team-sports, football, strength, change of direction ability, young athletes

## Abstract

The aim of this study was to compare changes in young soccer player's fitness after traditional bilateral vs. unilateral combined plyometric and strength training. Male athletes were randomly divided in two groups; both received the same training, including strength training for knee extensors and flexors, in addition to horizontal plyometric training drills. The only difference between groups was the mode of drills technique: unilateral (UG; *n* = 9; age, 17.3 ± 1.1 years) vs. bilateral (TG; *n* = 9; age, 17.6 ± 0.5 years). One repetition maximum bilateral strength of knee muscle extensors (1RM_KE) and flexors (1RM_KF), change of direction ability (COD), horizontal and vertical jump ability with one (unilateral) and two (bilateral) legs, and limb symmetry index were measured before and after an 8-week in-season intervention period. Some regular soccer drills were replaced by combination of plyometric and strength training drills. Magnitude-based inference statistics were used for between-group and within-group comparisons. Beneficial effects (*p* < 0.05) in 1RM_KE, COD, and several test of jumping performance were found in both groups in comparison to pre-test values. The limb symmetry index was not affected in either group. The beneficial changes in 1RM_KE (8.1%; *p* = 0.074) and 1RM_KF (6.7%; *p* = 0.004), COD (3.1%; *p* = 0.149), and bilateral jump performance (from 2.7% [*p* = 0.535] to 10.5% [*p* = 0.002]) were *possible* to *most likely* beneficial in the TG than in the UG. However, unilateral jump performance measures achieved *likely* to *most likely* beneficial changes in the UG compared to the TG (from 4.5% [*p* = 0.090] to 8.6% [*p* = 0.018]). The improvements in jumping ability were specific to the type of jump performed, with greater improvements in unilateral jump performance in the UG and bilateral jump performance in the TG. Therefore, bilateral strength and plyometric training should be complemented with unilateral drills, in order to maximize adaptations.

## Introduction

Soccer demands high levels of forceful and explosive movements such as heading, shooting and change of direction speed (Stølen et al., 2005), crucial to many game situations (Reilly et al., 2000) and decisive events during competition (Hoff and Helgerud, 2004). Improvements of strength and power may help athletes to improve short-duration maximal efforts during games, most likely contributing to competitive soccer performance (Wong et al., 2010). These traits should be trained independently from aerobic power with an optimal training stimulus (Helgerud et al., 2001), especially among young players during the in-season period (Ramírez-Campillo et al., 2014b, 2015a).

It is believed that by increasing muscular contraction force at high speed, explosive performance can be improved (Bangsbo, 1994). Stretch-shortening cycle muscle actions, such as those induced by plyometric training exercises—particularly as jump drills—do provide such training stimuli and are well-established techniques for enhancing athletic performance (Sáez de Villarreal et al., 2012), especially when combined with resistance training (Markovic and Mikulic, 2010; Meylan et al., 2014; Granacher et al., 2016). Plyometric exercises are widely believed to lead to positive adaptations in terms of power production and corresponding improvements in tasks strongly related with athletic performance in soccer (Arnason et al., 2004), such as maximal strength (Sáez-Sáez de Villarreal et al., 2010), jumping (Markovic, 2007), and change of direction speed (Asadi et al., 2016).

Bilateral-based and solely vertically oriented plyometric strength programs may not maximize training adaptations in young soccer players (Ramírez-Campillo et al., 2014b, 2015a,b). Training strategies must consider the unilateral and multiple-plane nature of most competitive soccer actions (Meylan et al., 2014), with players implicated in predominantly unilateral weight-bearing fundamental movements, such as running, cutting, kicking, vertical and horizontal leaps and changing running direction (Reilly, 1996; McCurdy et al., 2005). Moreover, this consideration is paramount, as soccer demands may impose on players various muscle strength asymmetries (Masuda et al., 2005). Strength asymmetries have been implicated with injuries to the lower limbs (Impellizzeri et al., 2007; Croisier et al., 2008) and may affect performance in tasks such as change of direction (Young et al., 2002). Thus, there is a need for soccer specific strength training interventions that incorporate multidirectional unilateral force production exercises.

Therefore, training programs should focus on unilateral (dominant and non-dominant leg) training to correct for asymmetries and to increase performance during recurrent unilateral actions during competition (Sinclair et al., 2014). However, relatively few studies have addressed this issue. Among females, greater improvements in power and jumping ability was observed after 6 weeks of unilateral plyometric training compared to bilateral training (Makaruk et al., 2011). In another study with males and females (McCurdy et al., 2005), unilaterally-trained subjects improved more than bilaterally-trained ones on unilateral relative power and vertical jump height. In a bilateral test, the improvements in power and jumping ability were similar in both groups. In young soccer players participating in plyometric-only training (Ramírez-Campillo et al., 2015a), an specific training change was observed, where unilateral-training induced greater performance gains in unilateral-dominant tests, whereas bilateral-training induced greater performance gains in bilateral-dominant tests. In a recently published study (Bogdanis et al., 2017) unilateral plyometric training was more effective at increasing jumping performance and maximal strength when compared to bilateral training. However, in the latter studies, plyometric training was not combined with resistance training. Since combination of strength and plyometric training may induce optimal adaptations compared with either training strategy alone (Lloyd et al., 2016), further research is required. In addition, since combination of strength and plyometric training is common among soccer teams (Bedoya et al., 2015; Wallenta et al., 2016; Behm et al., 2017), new research on the topic may offer findings of potential relevance for coaches, trainers, and scientists. Therefore, the purpose of this study was to compare the changes induced by traditional bilateral vs. unilateral combined plyometric and strength training in young soccer player's fitness. We hypothesized that both training approaches would induce beneficial changes in young soccer player's fitness, with improvements specific as the type of training program performed.

## Materials and methods

### Participants

Eighteen male young (U-19) soccer players with experience in soccer (≥8 years) and bilateral strength training (≥2 years) and from the same regional division team participated in this study. Athletes trained soccer on Monday, Wednesday, Thursday, and Friday, with one competition match on weekends. Soccer players have continually trained for the previous month with absence of musculoskeletal injury. Athletes were divided by simple randomization (Suresh, 2011) into a traditional bilateral strength and plyometric training group (TG; *n* = 9; age, 17.6 ± 0.5 years; height, 174.9 ± 5.3 cm; body mass, 68.3 ± 3.6 kg) and a unilateral group (UG; *n* = 9; age, 17.3 ± 1.1 years; height, 177.1 ± 5.9 cm; body mass, 64.9 ± 5.5 kg). One participant from each group withdrew from the study due to injury taking place during soccer competition. Although some athletes from both groups suffer minor sports trauma (Timpka et al., 2014) during the 8-week intervention, these occurrences did not involve withdraws, nor affected training adherence. The Ethics Committee of the Faculty of Education, Pontifical University of Salamanca, approved the study (Annex II of the Act November 22, 2017). Participants (and guardians for underage players) signed an informed consent document according to the Helsinki Declaration. Underage players provided assent.

The sample size was determined according to changes in vertical jumping performance in a group of soccer players subjected to a control (Δ = 0.5 cm; *SD* = 1.1) or a short-term plyometric training protocol (Δ = 2.6 cm; *SD* = 1.6) (Ramírez-Campillo et al., 2015a) comparable with that applied in this study. Eight participants per group would yield a power of 95% and α = 0.01.

### Experimental design

One repetition maximum strength of knee muscle extensors (1RM_KE) and flexors (1RM_KF), change of direction ability (COD), horizontal and vertical jump ability with one and two legs were measured before and after an 8-week in-season intervention period, where some regular soccer drills were replaced by combination of plyometric and strength training drills. All measurements were performed at the same venue, under identical conditions and by the same researchers, blinded for group's allocation. After a familiarization period, measurements were completed on 2 days separated by 48 h. During the first day the 1RM_KE, 1RM_KF and COD tests were performed. Jump tests were performed in the second day. To avoid measurements outcomes being affected by fatigue during jumping testing, the sequence of jump tests was counterbalanced. A 5-min general warm-up (self-paced jogging; skipping; strides; two acceleration runs) was performed before each testing day.

### Maximum strength tests

The bilateral 1RM_KE and 1RM_KF were measured in fitness machines (Reebok Fitness Machine®), following previous instructions (Titton and Franchini, 2017). After a standardized general warm-up, athletes perform 10 unloaded repetitions for each exercise, and then 5 repetitions with 50% of the perceived 10RM. After 3 min of rest, athletes completed the 10RM test. It was necessary just one attempt per athlete as they were familiarized with the test. The number of repetitions and the weight lifted were registered in order to obtain the 1RM_KE and the 1RM_KF, calculated from a previously validated equation (Brzycki, 1993). Due to logistical limitations, a unilateral testing procedure was implemented only at pre-intervention for the UG, in order to prescribe initial training loads.

### Change of direction ability test

**Figure 1:**
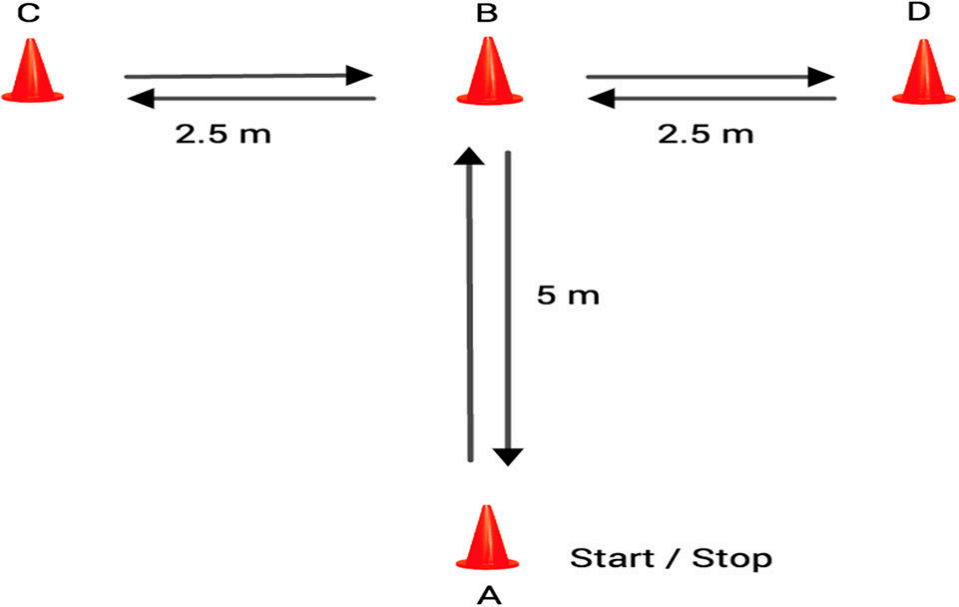
Modified agility *T*-test.

According to previous instructions (Sassi et al., 2009), athletes completed a modified agility *T*-test. A photocell gate system (DSD Laser System®) with its corresponding software (Sport Test, v3.2.1) was used to record the time. The players performed the test using the same directives as the traditional *T*-test, although they were not required to move laterally or face forward (Figure 1). The players had to touch the top of the cones instead of its base. A-B displacement (5-m): at his own discretion, each subject sprinted forward to cone B and touched the top of the cone with the right hand. B-C displacement (2.5-m): facing forward the participant shuffled to the left to cone C and touched the top of the cone with the left hand. C-D displacement (5-m): the soccer player then shuffled to the right to cone D and touched its top. D-B displacement (2.5-m): the participant shuffled back to the left to cone B and touched its top. B-A displacement (5-m): the soccer player moved as quickly as possible and returned to line A. Two maximal trials were completed and the best time was used for later analysis.

### Jumping tests and limb symmetry index

Athletes completed the countermovement jump (CMJ) and squat jump (SJ) tests following previous suggestions (Maulder and Cronin, 2005), with minimal flexion of the trunk during take-off (Blache and Monteil, 2015). The tests were also performed unilaterally, with dominant (CMJd and SJd) and non-dominant leg (CMJnd and SJnd). Limb dominance was detected by asking the player to kick a soccer ball with their preferred leg. Jumping was measured with a contact mat (Globus Ergo System®, Codogne, Italy). Athletes performed two maximal trials for each test with 1 min of rest in between. The maximal height achieved was selected for analysis.

According to previous validated protocols (Noyes et al., 1991; Rösch et al., 2000), athletes performed a horizontal triple jump with dominant (H3Jd) and non-dominant leg (H3Jnd). Briefly, athletes take three maximal jumps forward as far as possible on the testing leg and land on two legs during the final jump (Maulder and Cronin, 2005). Athletes also performed the horizontal triple jump with dominant and non-dominant leg using a cross-over (HC3Jd and HC3Jnd, respectively) pattern over a 15-cm marking strip, as previously described (Noyes et al., 1991). In short, athletes jumped three consecutive times using the dominant or non-dominant leg, crossing over the center strip on each hop. A bilateral horizontal CMJ with arms (HCMJ) was also performed, as a single jump maximal attempt but also as a triple jump maximal attempt (H3CMJ). For the six horizontal jumping tests, athletes performed two maximal trials, with a recovery of 3 min in between. The maximal distance achieved was selected for analysis. In all jumps, the hands were used freely. At the end of each horizontal jump attempt, athletes maintained the landing position for a brief moment.

As previously suggested (Gustavsson et al., 2006), the limb symmetry index was calculated after unilateral and bilateral jumping tests were completed, as: worse leg/better leg × 100.

### Training intervention

Athletes from both TG and UG groups maintained their regular soccer-training schedule during the 8-week in-season intervention period, including endurance training, small-sided games, tactical-technical training, friendly games, and injury prevention drills (no injuries associated to the training intervention were observed during the course of the study). Athletes from both training groups attended the same soccer training sessions as they belonged to the same regional division team. Therefore, soccer-training loads were equally distributed between TG and UG groups. However, the strength and plyometric training drills replaced some technical-warm up drills at the beginning of each intervention training session. The intervention was based on previous experience of the team's strength and conditioning coach and from previous reports (McCurdy et al., 2005; Makaruk et al., 2011; Ramírez-Campillo et al., 2015a). Athletes completed strength training on Wednesday and plyometric training on Wednesday and Friday. Between training sessions and competitions, a rest interval of ≥48 h was always allowed. Table 1 depicts the training program.

**Table 1:** Training program^*^.

**Wednesday**	**Friday**
**WEEK 1–4**	
Knee extensors 3 × 10 (70%) Knee flexors 3 × 10 (70%) 20-cm horizontal drop jumps^€^ 1 × 3–4 Horizontal jumps 1 × 3	20-cm horizontal drop jumps^€^ 1 × 3–4 Horizontal jumps 1 × 3
**WEEK 5–8**	
Knee extensors 3 × 10 (70%) Knee flexors 3 × 10 (70%) 25-cm horizontal drop jumps 1 × 4–5 Horizontal jumps 2 × 3	25-cm horizontal drop jumps 1 × 4–5 Horizontal jumps 2 × 3

* *: depicted exercises were performed bilaterally or unilaterally, for the traditional bilateral strength and plyometric training group (TG) and a unilateral group (UG), respectively*.

€ *: jump drills were performed with maximal voluntary effort*.

Both groups received the same training, volume per leg and coach to athlete supervision ratio. Following previous recommendations (Kraemer and Ratamess, 2004), and in line with the concept of minimal effective dose of training (Moran et al., 2017), during the strength sessions, athletes completed three sets of 10 repetitions for knee extensors and flexors muscles, at 70% 1RM_KE and 1RM_KF in bilateral (TG) or unilateral (UG) exercises. Unilateral 1RM_KE and 1RM_KF testing procedure were implemented only at pre-intervention for UG, in order to prescribe initial training loads. During the plyometric sessions, athletes completed one set of horizontal unilateral or bilateral drop jumps using 20-cm height boxes (10-cm height boxes for the UG) in the first 4 weeks and 25-cm height boxes (15-cm height boxes for the UG) in the last 4 weeks. Athletes completed three repetitions on week 1–3, four during weeks 4–6 and five in weeks 7–8. In addition to drop jumps, athletes completed a set of three consecutive horizontal jumps during the first 4 weeks, and two sets in the last 4 weeks. A low volume of training was deemed appropriate in order to soccer player cope with the rest of their soccer-related training and competitive activities. In addition, the volume applied was very similar to that used in previous effective strength and plyometric intervention with U20 young soccer players (Loturco et al., 2016), where nine jumps per session combined with resistance training induced meaningful improvements in sprint, COD and jumping performance. Each jump repetition was completed with maximal voluntary effort. Athletes were instructed to achieve maximal horizontal jump distance, with minimal ground contact time during drop jumps. Athletes had 2 min of rest between strength and plyometric training sets. Of note, both training groups completed the same total number of sets and repetitions per leg. Each session lasted ~15 min. Athletes were asked to attend >80% all training sessions during the intervention to be included in the final analysis.

### Statistical analysis

Data are presented as mean ± standard deviation (SD). A Shapiro-Wilk test was used to analyse the normally distributed data. Firstly, a traditional null-hypothesis testing was conducted. Within-group comparisons (Student paired *t*-test) were carried out to detect significant differences between the pre-test and post-test in any variable in both groups. In addition, an ANCOVA (general linear model) was used to detect any significant between-group difference at post-test using the pre-test as a covariate (IBM SPSS Statistics 21, IBM Co., USA). Thereafter, all data were log-transformed to reduce bias arising from non-uniformity error (magnitude-based inferences approach). The standardized difference or effect size (ES, 90% confidence limits [CL]) in the selected variables was calculated using the pooled pre-training SD. Threshold values for Cohen's ES statistics were >0.2 (small), >0.6 (moderate), and >1.2 (large) (Hopkins et al., 2009). For within/between-group comparisons, the chances that the differences in performance were better/greater [i.e., greater than the smallest worthwhile change (0.2 multiplied by the between-subject standard deviation, based on Cohen's d principle)], similar or worse/smaller were calculated. Quantitative chances (QC) of beneficial/better, similar/trivial or detrimental/poorer effect were assessed qualitatively as follows: <1%, almost certainly not; >1–5%, very unlikely; >5–25%, unlikely; >25–75%, possible; >75–95%, likely; >95–99%, very likely; and >99%, most likely (Hopkins et al., 2009). If the chance that the true value is >25% beneficial and >0.5% chance that it is harmful, the clinically effect was considered as unclear. However, the clinical inference was declared as beneficial when odds of benefit/harm was >66 (Hopkins et al., 2009). Two specific Excel spreadsheets from sportsci.org were used to examine both the between-group (xCompare2groups.xls) and within-group (xPostOnlyCrossover.xls) comparisons.

## Results

### Within-group changes

Beneficial changes in 1RM_KE (TG: 8 out of 9 individual improvements; UG: 4 out of 9 individual improvements), COD (TG: 9/9; UG: 6/9), CMJ (TG: 8/9; UG: 6/9), CMJd (TG: 5/9; UG: 8/9), CMJnd (TG: 4/9; UG: 7/9), SJnd (TG: 3/9; UG: 6/9), and HC3Jnd (TG: 4/9; UG: 7/9) were found in both groups in comparison to pre-test values. In the TG, beneficial gains were reported 1RM_KF (6 out of 9 improvements), SJ (9/9) and HCMJ (9/9). In the UG SJd (8/9), H3Jd (8/9), H3Jnd (8/9), and HC3Jd (8/9) were likely to very likely improved (Table 2). The limb symmetry index was not affected in either group. Furthermore, significant differences in each group were reported in Table 2.

**Table 2:** Changes in athletic performance following bilateral combined plyometric and strength training (*n* = 9) or unilateral combined plyometric and strength training (*n* = 9). Data are mean ± SD.

	**Bilateral (*n* = 9)**						**Unilateral (*n* = 9)**					
	**Pre-test**	**Post-test**	**% (CL90%)**	**ES (CL90%)**	**Chances**	**Outcome**	**Pre-test**	**Post-test**	**% (CL90%)**	**ES (CL90%)**	**Chances**	**Outcome**
1RM_KE (kg)	207.9 ± 30.2	237.2 ± 51.0^*^	12.9 (7.4; 18.6)	0.77 (0.46; 1.09)	100/0/0%	Most Likely	179.8 ± 9.1	182.4 ± 8.4^*^	1.5 (0.4; 2.6)	0.27 (0.07; 0.47)	73/27/0%	Possibly
1RM_KF (kg)	93.8 ± 11.2	99.8 ± 10.2^*^	6.6 (2.2; 11.2)	0.48 (0.16; 0.80)	93/7/0%	Likely	86.5 ± 2.9	86.8 ± 2.9	0.3 (−0.3; 1.0)	0.09 (−0.08; 0.25)	12/87/1%	Likely trivial
COD (s)	5.67 ± 0.29	5.46 ± 0.20^*^	3.7 (1.3; 6.0)	0.66 (0.23; 1.09)	96/4/0%	Very Likely	5.80 ± 0.29	5.67 ± 0.16	2.2 (−0.7; 5.1)	0.41 (−0.13; 0.96)	76/21/4%	Likely
CMJ (m)	0.37 ± 0.05	0.40 ± 0.05^*^	8.6 (4.2; 13.1)	0.56 (0.28; 0.84)	98/2/0%	Very Likely	0.38 ± 0.04	0.40 ± 0.05	5.2 (−0.8; 11.6)	0.51 (−0.08; 1.11)	82/15/3%	Likely
CMJd (m)	0.22 ± 0.03	0.22 ± 0.03	2.8 (0.1; 5.6)	0.18 (0.01; 0.35)	41/59/0%	Possibly	0.23 ± 0.03	0.26 ± 0.03^*^	10.0 (6.9; 13.1)	0.85 (0.59; 1.10)	100/0/0%	Most Likely
CMJnd (m)	0.20 ± 0.03	0.20 ± 0.02	2.7 (−0.5; 6.0)	0.19 (−0.03; 0.42)	47/52/1%	Possibly	0.22 ± 0.02	0.24 ± 0.03^*^	6.4 (2.1; 10.9)	0.50 (0.17; 0.83)	93/6/0%	Likely
LSI_CMJ (%)	90.8 ± 4.6	90.7 ± 4.8	−0.1 (−4.0; 3.8)	−0.02 (−0.71; 0.66)	28/40/32%	Unclear	91.9 ± 6.3	91.1 ± 6.8	−0.8 (−3.2; 1.6)	−0.11 (−0.42; 0.20)	5/65/30%	Possibly harmful
SJ (m)	0.35 ± 0.05	0.37 ± 0.04^*^	6.5 (2.6; 10.6)	0.36 (0.15; 0.58)	90/10/0%	Likely	0.36 ± 0.05	0.34 ± 0.04	−6.0 (−12.8; 1.2)	−0.45 (−0.99; 0.09)	3/18/79%	Likely harmful
SJd (m)	0.21 ± 0.03	0.21 ± 0.03	1.1 (−1.4; 3.6)	0.08 (−0.11; 0.26)	12/87/1%	Likely trivial	0.22 ± 0.02	0.24 ± 0.02^*^	8.1 (3.1; 13.2)	0.64 (0.25; 1.02)	97/3/0%	Very Likely
SJnd (m)	0.18 ± 0.02	0.19 ± 0.02	3.1 (−0.1; 6.5)	0.26 (−0.01; 0.53)	66/33/1%	Possibly	0.22 ± 0.03	0.23 ± 0.03^*^	4.8 (1.3; 8.5)	0.35 (0.09; 0.60)	84/15/0%	Likely
LSI_SJ (%)	87.7 ± 7.9	88.6 ± 9.2	1.0 (−1.3; 3.3)	0.09 (−0.13; 0.32)	20/78/2%	Likely trivial	91.5 ± 8.4	89.8 ± 3.1	−1.5 (−6.3; 3.6)	−0.14 (−0.61; 0.33)	11/48/41%	Possibly harmful
H3Jd (m)	6.49 ± 0.30	6.53 ± 0.28	0.5 (−0.2; 1.2)	0.10 (−0.04; 0.24)	11/89/0%	Likely trivial	6.19 ± 0.32	6.62 ± 0.31^*^	7.1 (3.7; 10.5)	1.21 (0.64; 1.78)	99/0/0%	Very Likely
H3Jnd (m)	6.29 ± 0.30	6.29 ± 0.31	0.0 (−0.4; 0.3)	−0.01 (−0.07; 0.05)	0/100/0%	Most Likely trivial	5.97 ± 0.33	6.35 ± 0.34^*^	6.4 (2.7; 10.3)	0.99 (0.42; 1.55)	98/1/0%	Very Likely
LSI_H3J (%)	96.8 ± 4.2	96.1 ± 3.9	−0.7 (−1.4; 0.0)	−0.14 (−0.28; 0.00)	0/78/22%	Likely trivial	96.5 ± 2.8	95.2 ± 3.3	−1.4 (−3.8; 1.1)	−0.43 (−1.20; 0.34)	8/21/71%	Possibly harmful
HC3Jd (m)	5.62 ± 0.40	5.70 ± 0.39	1.5 (−1.9; 4.9)	0.17 (−0.23; 0.57)	45/49/6%	Unclear	5.66 ± 0.42	5.98 ± 0.38^*^	5.8 (1.7; 10.0)	0.65 (0.20; 1.10)	95/5/0%	Likely
HC3Jnd (m)	5.49 ± 0.47	5.71 ± 0.71	4.0 (−0.8; 9.1)	0.39 (−0.08; 0.87)	76/21/2%	Likely	5.28 ± 0.56	5.70 ± 0.45^*^	8.2 (2.1; 14.6)	0.64 (0.17; 1.11)	94/6/0%	Likely
LSI_HC3J (%)	95.6 ± 1.6	95.7 ± 2.4	0.1 (−1.7; 1.9)	0.04 (−0.95; 1.03)	39/28/33%	Unclear	93.3 ± 5.9	95.3 ± 3.8	2.2 (−2.5; 7.2)	0.30 (−0.34; 0.94)	61/30/9%	Unclear
HCMJ (m)	2.24 ± 0.08	2.34 ± 0.07^*^	4.5 (2.5; 6.5)	1.17 (0.67; 1.67)	100/0/0%	Most Likely	2.20 ± 0.15	2.21 ± 0.15	0.7 (0.1; 1.3)	0.09 (0.01; 0.17)	2/98/0%	Very unlikely
H3CMJ (m)	6.56 ± 0.58	6.65 ± 0.56^*^	1.3 (0.5; 2.2)	0.13 (0.05; 0.21)	8/92/0%	Unlikely	6.67 ± 0.58	6.84 ± 0.52	2.7 (−2.1; 7.6)	0.28 (−0.23; 0.79)	62/33/6%	Unclear

1RM_KE, one-repetition maximum in knee extensors; 1RM_KF, one-repetition maximum in knee flexors; COD, modified agility test; CMJ, bilateral countermovement jump; CMJd and CMJnd, unilateral countermovement jump with dominant and non-dominant leg; LSI_CMJ, limb symmetry index in unilateral countermovement jump; SJ, bilateral squat jump; SJd and SJnd, unilateral squat jump with dominant and non-dominant leg; LSI_SJ, limb symmetry index in unilateral squat jump H3Jd and H3Jnd, horizontal triple jump with dominant and non-dominant leg; LSI_H3J, limb symmetry index in unilateral horizontal triple jump; HC3Jd and HC3Jnd, crossover horizontal triple jump with dominant and non-dominant leg; LSI_HC3J, limb symmetry index in unilateral crossover triple jump; HCMJ, bilateral horizontal jump with arm swing; H3CMJ, triple bilateral horizontal jump with arm swing. Significant differences (p < 0.05) between pre-test to post-test were indicated with ^*^.

### Between-group changes

**Figure 2:**
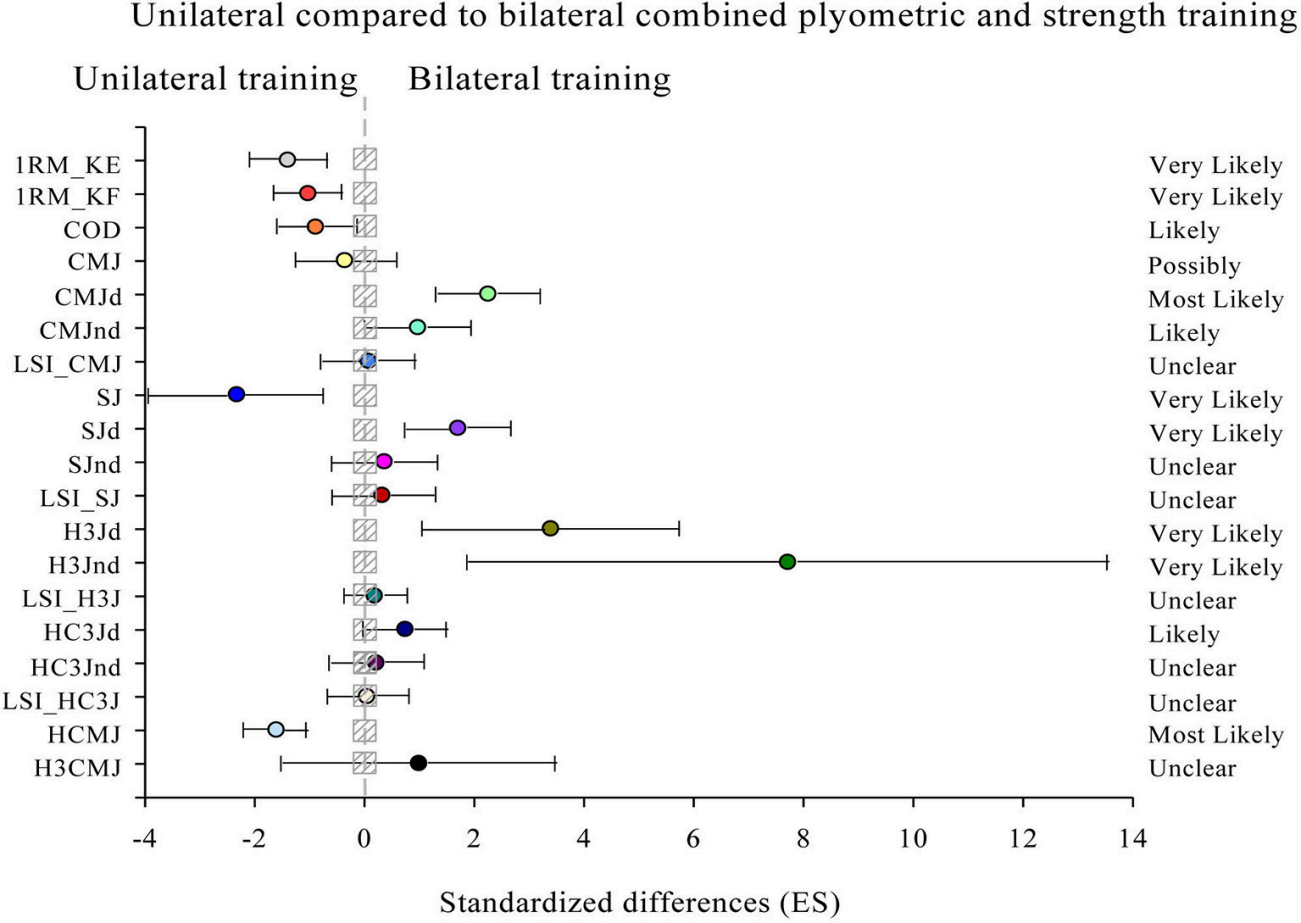
Efficiency of the unilateral (UNI) compared to bilateral (BIL) combined plyometric and strength training to improve one-repetition maximum in knee extensors (1RM_KE), one-repetition maximum in knee flexors (1RM_KF), a modified agility test (COD), bilateral countermovement jump (CMJ), unilateral countermovement jump with dominant and non-dominant leg (CMJd and CMJnd), limb symmetry index in unilateral countermovement jump (LSI_CMJ), bilateral squat jump (SJ), unilateral squat jump with dominant and non-dominant leg (SJd and SJnd), limb symmetry index in unilateral squat jump (LSI_SJ), horizontal triple jump with dominant and non-dominant leg (H3Jd and H3Jnd), limb symmetry index in unilateral horizontal triple jump (LSI_H3J), crossover horizontal triple jump with dominant and non-dominant leg (HC3Jd and HC3Jnd), limb symmetry index in unilateral crossover triple jump (LSI_HC3J), bilateral horizontal jump with arm swing (HCMJ), and triple bilateral horizontal jump with arm swing (H3CMJ) (bars indicate uncertainty in the true mean changes with 90% confidence limits). Trivial areas were the smallest worthwhile change (SWC) (see Methods).

Results from between-group analyses are illustrated in Figure 2. The beneficial changes in 1RM_KE (8.1% [CL90%: 4.2; 11.8]; *QC* = 99/0/0%; *p* = 0.074) and 1RM_KF (6.7% [CL90%: 2.8; 10.4]; *QC* = 98/1/0%; *p* = 0.004), COD (3.1% [CL90%: 0.5; 5.7]; *QC* = 94/5/1%, *p* = 0.149), CMJ (2.7% [CL90%: −4.8; 9.7]; *QC* = 61/23/16%; *p* = 0.535), SJ (10.5% [CL90%: 3.5; 17.0]; *QC* = 98/1/1%; *p* = 0.013) and HCMJ (4.4% [CL90%: 2.7; 6.0]; *QC* = 100/0/0%; *p* = 0.002) were possibly to most likely beneficial in the TG than in the UG. However, CMJd (8.4% [CL90%: 4.7; 12.1]; *QC* = 100/0/0%; *p* = 0.006), CMJnd (5.9% [CL90%: 0.0; 12.1]; *QC* = 91/6/3%; *p* = 0.081), SJd (8.6% [CL90%: 3.6; 13.8]; *QC* = 99/1/0%, *p* = 0.018), H3Jd (4.8% [CL90%: 1.4; 8.2]; *QC* = 98/1/1%, *p* = 0.023), H3Jnd (5.0% [CL90%: 1.2; 8.9]; *QC* = 98/2/0%; *p* = 0.034) and HC3Jd (4.5% [CL90%: 0.1; 9.1]; *QC* = 89/9/2%; *p* = 0.090) achieved likely to most likely beneficial changes in the UG compared to the TG.

## Discussion

The purpose of this study was to compare the changes induced by traditional bilateral vs. unilateral combined plyometric and strength training in young soccer player's fitness. We hypothesized that both training approaches would induce beneficial changes in young soccer player's fitness, with improvements specific to the type of training program performed. Main findings indicated that both groups improved 1RM_KE, COD and jumping ability, with no changes in limb symmetry index. A specificity was noticed for adaptations, whereby only the TG improved the bilateral maximal strength of the knee flexors and the bilateral jump performance in the SJ and HCMJ, and only the UG improved unilateral performance in the SJd, H3Jd, H3Jnd, and HC3Jd tests. Moreover, improvements in 1RM_KE and 1RM_KF, COD, CMJ, SJ, and HCMJ were greater in the TG than in the UG, while improvements in CMJd, CMJnd, SJd, H3Jd, H3Jnd, and HC3Jd were greater in the UG. Current results are in line with our hypothesis and corroborate previous findings (Behm et al., 2017) related to the efficacy of combined strength and plyometric training in young soccer players, and its specific effects.

Both trained groups improved 1RM_KE, and the magnitude of the improvement is in line with previous findings (Granacher et al., 2016). As previously suggested the specific strength training probably explains most of the improvement (Behm et al., 2017). However, plyometric training might also help to explain the performance gains (Sáez-Sáez de Villarreal et al., 2010). Muscle strength has been significantly correlated with soccer player's sprint, COD and jumping ability (Wisløff et al., 2004). Considering that sprinting, COD and jumping actions are highly demanded during soccer competition (Reilly et al., 2000; Hoff and Helgerud, 2004; Stølen et al., 2005) and are related to team success (Arnason et al., 2004), it might be possible that such strength improvements play a decisive difference during competition. Of note, only the TG improved 1RM_KF. Moreover, the 1RM_KE and 1RM_KF muscles' improvements were greater in the TG compared to the UG. However, these differences might have been artificially created through the implementation of a bilateral maximum strength testing procedure. Due to logistical limitations, the unilateral testing procedure was implemented only at pre-intervention for the UG, in order to prescribe initial training loads. Future studies should seek to clarify this issue.

Regarding COD ability, both trained groups improved performance. Both strength (Sheppard and Young, 2006; Young and Farrow, 2006) and plyometric training (Asadi et al., 2016) might have contributed to improved COD performance. Previous studies have also found COD improvements after strength-plyometric training interventions with young soccer players (Ramirez-Campillo et al., 2014a; Ramírez-Campillo et al., 2014b; Bedoya et al., 2015; Kobal et al., 2017). Improvements in power (Negrete and Brophy, 2000), reactive strength (Young et al., 2002), eccentric strength (Sheppard and Young, 2006), as well as maximal strength (Rouissi et al., 2016) may help explain the COD improvement. Of note, the TG had a likely greater COD improvement compared to the UG. This result contrasts with previous findings, where greater improvements in unilateral-related performance actions were observed after unilateral training programs (McCurdy et al., 2005; Makaruk et al., 2011; Bogdanis et al., 2017). Moreover, current results contrast with a previous study (Ramírez-Campillo et al., 2015a) where young soccer players achieved greater COD improvement after unilateral compared to bilateral plyometric training. It might be possible that the use of different COD test and training programs between studies partially explain the difference. In addition, in the current study only horizontal plyometric drills were used, whereas the combination of both vertical and horizontal drills might optimize adaptations (Ramírez-Campillo et al., 2015b). Considering the unilateral nature of most competitive soccer actions (Meylan et al., 2014), including COD (Reilly, 1996; McCurdy et al., 2005), we deemed prudent to recommend the inclusion of unilateral drills in young soccer training programs.

Current results indicate favorable changes in jumping performance in both training groups. Jumping ability may be considered an independent physical attribute related to soccer performance (Arnason et al., 2004). Further, jumping ability may indirectly positively affect other key physical attributes such as sprint acceleration (Nikolaidis et al., 2016), which may be applicable to players of different positions (Nikolaidis et al., 2014). Although both training groups improved CMJ, CMJd, CMJnd, SJnd, and HC3Jnd, only the TG improved SJ and HCMJ, whereas only the UG improved SJd, H3Jd, H3Jnd, and HC3Jd test results. Moreover, improvements in CMJ, SJ and HCMJ were greater in the TG, whereas CMJd, CMJnd, SJd, H3Jd, H3Jnd, and HC3Jd improvements were greater in the UG. Although the specificity of training is a well-established training principle (Behm et al., 2017), few studies have corroborated this phenomenon in young soccer players, especially after unilateral and bilateral plyometric and strength training approaches (Ramírez-Campillo et al., 2015a). In this sense, replication studies are necessary to increase generalization of findings, with current results aiding to this aim. Motor coordination adaptations may be related to the specificity of movements used during training (Diallo et al., 2001), therefore the changes induced by either unilateral or bilateral jump training drills are higher for actions in which athletes have been specifically trained. Therefore, if athletes require specific unilateral of bilateral improvements in some soccer-related action, coaches should strive to allocate a greater proportion of appropriate drills into the athlete's training schedule. Moreover, current specificity-related results are of paramount importance from a transference point of view, since more specific training approaches offer more transference toward specific performance aims (Loturco et al., 2016). Therefore, current results offer novel results not only from a specificity point of view, but also from a transference point of view.

The limb symmetry index or the between-limbs imbalance is a valid and useful tool to detect players at high risk of lower-limb injury [i.e., 4-fold in players with >10% of asymmetry (Gustavsson et al., 2006), as well as to a successful return to sport after an ACL injury (Ardern et al., 2012)]. Furthermore, functional asymmetries might play a key role in performance (Maloney et al., 2017). To our knowledge, the only study that has analyzed the change of an intervention on limb symmetry index (Gonzalo-Skok et al., 2017) shows that unilateral training might prove effective in reducing between-limb imbalance. However, neither within group nor between-group differences were found in the current study. Therefore, no significant differences in improvements for the dominant compared to the non-dominant leg were observed over time. Between-studies differences might be due to the strength training developed (single-joint vs. multi-joint exercise), the number of repetitions performed (pre-determined vs. post-determined), the athletes' level (regional vs. national/international) or the exercise used to analyse the limb symmetry index. The contrasting changes open a research window to improve our understanding of the effects of unilateral training on reducing the between-limbs differences, and its potential on injury prevention.

As practical recommendations, the replacement of some low-intensity soccer drills with strength and maximal-intensity plyometric drills during the warm-up may positively affect jumping, changing of direction ability and strength during the in-season, even in well trained young soccer players. These improvements might aid performance in competition and may reduce injury risk (Arnason et al., 2004). Current results indicate that bilateral training offer advantages to improve COD, bilateral strength and jumping performance, while unilateral training induced greater gains in unilateral jumping. However, considering the unilateral nature of most competitive soccer actions, including COD (Rouissi et al., 2016), and to maximize adaptations among young soccer players (Ramírez-Campillo et al., 2015a), it is recommended that during training sessions soccer players combine unilateral and bilateral drills, executed in different planes (Meylan et al., 2014; Ramírez-Campillo et al., 2015b). However, if unilateral or bilateral movements are particularly important for the athlete, due to the specificity of adaptations observed in current study, a high portion of drills should be executed with the required movement pattern. Moreover, practitioners may recommend targeting the worse leg more than the better leg in unilateral training to reduce asymmetry between legs. More research is needed in order to better understand the changes of unilateral training on reducing the between-limbs differences (limb symmetry index), and its potential on injury prevention. Of note, in current study soccer players had extensive experience with strength training. Therefore, in order to better replicable current findings with soccer players, an adequate foundation of strength is advised before introducing plyometric drills (Behm et al., 2017).

A potential limitation of the current study is the lack of a control group. However, in previous seasons soccer players of current study had used the bilateral training program, with significant results. In addition, considering the specific adaptations observed in both experimental groups, it seems evident that the observed changes may be explained by the specific training interventions. However, future studies should aim to replicate current results with a controlled study design, including replication with females, and a greater number of participants. Another potential limitation is related with the bilateral deficit-potentiation phenomenon (Škarabot et al., 2016). In this sense, it may have been possible that different amounts of work-power were completed by the bilateral vs. unilateral trained groups, affecting the outcomes of our study. However, when values of jumping performance (SJ, CMJ) in Table 2 are considered, when jumping with both legs subjects achieved almost twice the work (body mass × height) compared to jumping with one leg. In this sense, unilaterally and bilaterally trained athletes probably achieved similar values of total work. Of note, due to the large number of comparisons in our study, the risk of type-1 error may have been increased. Although *p*-value adjustments may have reduced the chance of making type I errors (Feise, 2002), this may have increased the chance of making type II errors. Therefore, we also include an ES analysis. In our view, this allows a more comprehensive perspective of the results.

To conclude, both training groups improved maximal strength of knee extensors, change of direction and jumping ability, with no changes in limb symmetry index. The improvements in the maximal strength of knee extensors and flexors, and the change of direction ability were beneficially greater in the traditional bilateral group vs. the unilateral combined strength and plyometric training group. The improvements in jumping ability were specific to the type of jump performed, with greater improvements in unilateral jump in the unilateral training group and bilateral jump performance in the bilateral training group. Therefore, bilateral strength and plyometric training should be complemented with unilateral drills, in order to maximize adaptations throughout the season.

## Author contributions

RR-C and JS-S: Designed the work; MC, AR-F, and OG-S: Data acquisition; FN, RR-C, and JS-S: Analysis and interpretation of data; drafting the work; RR-C, JS-S, OG-S, MC, AR-F, and FN: Revising critically the work; final approval of the version to be published; agree to be accountable for all aspects of the work in ensuring that questions related to the accuracy or integrity of any part of the work were appropriately investigated and resolved.

### Conflict of interest statement

The authors declare that the research was conducted in the absence of any commercial or financial relationships that could be construed as a potential conflict of interest.
